# Investigating the availability of 3D-printed firearm designs on the clear web

**DOI:** 10.1016/j.fsisyn.2023.100444

**Published:** 2023-10-18

**Authors:** Stefan Schaufelbühl, Aurélie Szwed, Alain Gallusser, Olivier Delémont, Denis Werner

**Affiliations:** a Ecole des Sciences Criminelles, University of Lausanne, Switzerland; b National Institute of Criminalistics and Criminology, Brussels, Belgium; c Department of Chemistry, Biochemistry and Physics, Université du Québec à Trois-Rivières, Canada; d Groupe de Recherche en Science Forensique (GRSF), 3351 Boulevard des Forges, Trois-Rivières, Québec G9A 5H7, Canada

**Keywords:** Additive manufacturing, Blueprint, Fully 3D-printed firearms (F3DP), Hybrid firearms, Parts kit completions / conversions (PKC)

## Abstract

The release of the plans of the 3D-printed Liberator firearm sparked a wave of new designs from creators worldwide, resulting in an extensive collection of 3D-printed firearm plans, in particular blueprints, and parts available for almost unrestricted download on the internet. Identifying and categorizing the diverse range of 3D-printed firearms and components pose a challenge due to the abundance of designs available. Between 2021 and April 2023, data was collected on over 2,100 3D-printed firearm plans. While blueprints of fully 3D-printed firearms initially dominated the scene, hybrid designs and parts kit completions / conversions (PKC) have gained popularity for their improved reliability and performance. The now highly networked community offers considerable support with detailed instructions and procedures, providing precise guidance for construction. This systematic classification, grouping and structuration of the recorded data on the Clear Web supported the identification of patterns of the main threat trends related to 3D-printed firearms.

## Highlights

• Data on over 2,100 3D-printed firearm plans was gathered from the internet.
• An overall increase in hybrid firearm and PKC build activity has been noted.
• Many blueprints of Glock pistol frames and AR-15 lower receivers have been observed.
• 3D-printed firearm blueprints are mostly designed for handgun calibre ammunition.
• A small core of people dominates the online activity behind 3D-printed firearms.

## 1 Introduction

Additive Manufacturing (AM), widely known as 3D printing, has been developing and democratising in our society since its inception in 1986, so much so that, today, it is a reliable process that can be used by everyone. Indeed, the technology allows the production of objects that may have complex geometry, for example through the application layer-by-layer of a chosen material, according to computer-aided design model data [1]. It does not only allow a wide freedom of design but is also a sustainable technology which has the potential to reduce the global amount of carbon emissions, the development costs and time to market altogether [2]. Showing such promises, the AM market currently has an annual growth rate of more than 20 % [3].

In May 2013, the blueprints of a fully 3D-printed (F3DP) firearm, the Liberator, were uploaded on the internet by Cody Wilson, founder of Defense Distributed, a community aiming at producing and sharing such files. Alongside these blueprints, Cody Wilson also uploaded a video of himself successfully firing a shot with the assembled 3D-printed firearm. Then, this F3DP firearm was tested subsequently by several law enforcement agencies and studies concluded that it could be considered functional and dangerous, both for the user and the potential target [[4], [5], [6], [7]]. Upon release of the Liberator's blueprints a legal debate was engaged between the American authorities and Defense Distributed, but also worldwide surrounding the concept of those 3D-printed firearms. In most countries, 3D-printed firearm access and possession are strictly regulated, but the free distribution of plans and blueprints for 3D-printed firearms remains still nowadays in a grey zone, more often than not. Since then, following Defense Distributed's lead, several communities aimed at pursuing the development of 3D-printed firearm blueprints, in whole or in parts, have emerged on the internet and especially on the Clear Web. Groups such as FOSSCAD (Free Open-Source Software – Computer Aided Design) or Deterrence Dispensed operate as collectives of 3D-printed firearms designers and publishers, navigating and communicating across multiple platforms on the internet. They often share pre-release files and blueprints of 3D-printed firearms for peer review and testing, thus allowing them to improve their design to the final release [8].

The manufacturing of 3D-printed firearms by AM encompasses a variety of different products. Conventionally, three categories of 3D-printed firearms are differentiated: (i) fully 3D-printed (F3DP) firearms, (ii) hybrid firearms and (iii) parts kit completions / conversions (PKC) firearms [8]. F3DP firearms consist mainly of 3D-printed parts, apart from the firing pin, generally a manufactured nail, and sometimes elastic bands to drive parts. An example of this is the aforementioned Liberator, PM422 Songbird, PM522 Washbear, Marvel Revolver and Grizzly [9]. Hybrid firearms are largely made from 3D printing but have essential non-printed and mostly non-regulated parts integrated in their design. Probably the best-known hybrid firearm is the FGC-9, which uses amongst other metallic parts, such as a metal tube acting as a barrel and an AR-15 or modified airsoft trigger system. The last category, the PKC, includes 3D-printed firearms that consist of a 3D-printed receiver or frame which is then assembled with components from conventional firearms, as for example, frames for Glock pistols or lower receivers for AR-15 [8]. Comparing the three categories, the reliability and capacities of the 3D-printed firearms increase from F3DP firearms via hybrid to PKC designs. However, in the same order, the complexity of the models, the skills needed to produce them, and the costs also increase.

An article published by Listek [9] shows that law enforcement is already dealing with this category of firearm. It reveals that in the last three years alone, almost 100 people have been arrested for crimes involving 3D-printed firearms worldwide. The trend has been showing a steep increase for the past few years. Also, in a communication published by Europol in 2022, it was confirmed that these categories of homemade firearms are on the rise in Europe and that more attention should be focused on them [10]. One problem is that the 3D-printed firearms are constantly being developed, resulting in new models every year. In the face of this rapid development, published scientific studies (criminological, forensic, etc.) are scarce, and they struggle to produce up-to-date information on the mechanisms and extent of the phenomenon. The result is a knowledge gap that needs to be filled if law enforcement agencies are to be properly aware of this challenge and deal with it.

This concerns, first and foremost, the basic knowledge of the variety of 3D-printed firearms that are available, as well as the accessibility on the internet of the plans, in particular blueprints, to produce them. We therefore decided to take stock of the situation and provide an overview of the different 3D-printed firearm models available. For this purpose, we have carried out a review of the sources providing files of the three categories of 3D-printed firearms on the Clear Web. The data obtained was computed to establish trends around these designs and to provide information which can be used in the investigation of future casework related to the use of these 3D-printed firearms.

## 2 Methodology

### 2.1 Information gathering

As mentioned earlier, it appears that a lot of information on 3D-printed firearms and their plans, in particular blueprints, are readily available on the Clear Web which is a set of web spaces sharing resources located by URLs; but also on the so-called Dark Web, a part of the Web not indexed by web search engines nor regulated, and that requires a specific communication protocol to access it [11]. As a result, this research relies on data collected on a range of websites on the Clear Web, which were all publicly available. Websites of interest were identified through available search engines as well as social media (see Table 1). As soon as files were found, information about the site was collected and all relevant files were downloaded if they were available free of charge. On sites with paid access to the files, only the free information available on the web page itself was collected and recorded. The information gathering was done over a period of time between the start of 2021 and April 2023 using a Windows 10 Computer with Google Chrome (different versions).

**Table 1:** Websites utilised during the research on the Clear Web.

Search engines	Social media
Google Search	Reddit
Bing	Twitter
Yahoo!	Youtube
DuckDuckGo	Facebook

### 2.2 Data collection and exploitation

All the obtained data was collated and structured in a Microsoft Excel file.^1^ For each new entry (row in the table), the following variables (column headings) were collated: *Blueprint ID, Website ID, Pathway ID*, *Number of entry, URL, Model name*, *Category of 3D-printed firearm*, *Calibre*, *Main PoI* (Person of Interest), *Publication date*, *Other PoI* and *Comments*.

**Fig. 1:**
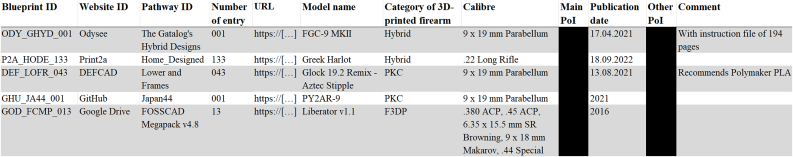
Screenshot of the Excel sheet used for data collection. Note that the cells marked in black were made to anonymise names or pseudonyms of the individuals active in this domain.

The *Blueprint ID* is a unique indexation number assigned to each blueprint of a 3D-printed firearm design recorded from each source. This ID is composed of a three-digit abbreviation of the website, pathway, and the number assigned to a blueprint of a 3D-printed firearm model. *Website ID* as well as *Pathway ID* describe the origin of the collected data. Each website may include various *Pathway IDs* (e.g., user accounts, repositories on GitHub, folder paths on file sharing sites). For example, GitHub.com is a website that has been assigned a *Website ID* for the purpose of this research. The repositories of different users discovered on this website have then been assigned different *Pathway IDs*, allowing the origin of the data to be more clearly identified within a website. In the following sections, the term “source” refers to the origin of data in a generic meaning and thus includes *Website ID* as well as *Pathway ID*. The *number of entry* assigns each model a unique number within a source. The *URL* is the direct link to the source of a certain blueprint of a 3D-printed firearm design, which was valid at the time of capture. These links are subject to change over time, for various reasons (e.g., deletion of file, changes of the source, etc.). The *Model name* refers to the 3D-printed firearm's designation on the source. The *Category of 3D-printed firearm* and *Calibre* describe the category of the 3D-printed firearm (e.g., F3DP, Hybrid or PKC) and the calibre(s) that can be fired from it. As the classification between F3DP and hybrid firearms can be unclear, it was decided that F3DP firearms are only considered as such if all essential parts of the 3D-printed firearm, such as the barrel, frame and breech, are printed, and only a few non-printed parts that are needed for their basic functioning (e.g., a nail as a firing pin or rubber bands to operate the moving parts). 3D-printed firearms containing non-printed parts that are intended to increase reliability (e.g., metal tubes as barrels or other reinforcements) were classified as hybrids. *Main PoI* and *Publication date* refers to the user on the internet who designed or published the 3D-printed firearm blueprint and on which date. Due to lack of information and access to some information, it was not always possible to attribute an exact designer, to differentiate between designer and publisher or to determine the date of publication. Nevertheless, this data provides information about the activity of a person and on the source. For example, this shows when a Main PoI has published a certain design on different sources and thus also proves its activity across various sources. *Other PoI* refers to people that collaborated with the project. Lastly, the section *Comments* contains further relevant information in free text format. A cropped section of the Excel file is shown in Fig. 1.

In order to highlight trends or cross reference information, pivot and contingency tables were made using Microsoft Excel functionalities. This allows you to display relevant values in relation to different variables that may be dependent on one another.

## 3 Results and discussion

### 3.1 Census of information

A total of 2,133 entries were collated in the Excel table on 3D-printed firearm blueprints. The number of *Website IDs* accounts to 7 (i.e., Cults3D, DEFCAD, GitHub, Google Drive, Odysee, Print2a and Thingiverse) while the *Pathway IDs* total at 45. Identifying sources was mainly done via the FOSSCAD subreddit on which information about 3D-printed firearms as well as any other related matter can be found [12]. This subreddit is comprised of around 74,000 members in early 2023. Additional sources were identified by browsing through Twitter accounts of well-known members of the 3D-printed firearms community. Nevertheless, some of the sources were also identified through different search engines available on the internet.

The collation of data was sometimes made difficult because of differences in information from one source compared to another for the same blueprint of a 3D-printed firearm design. It is common occurrence that sources are deleted, and that files are moved to another site subsequently. Such changes may be due to policy changes by the site owners or due to new requirements that the current source does not offer. In addition, the relatively large number of *Pathway IDs* made it difficult to manually keep track of them, and it emerged that there was no standardised method of file-sharing. Moreover, this community has a mindset that approves of sharing and modifying files. The aim for the community is to gain more reach on the various platforms, and for existing designs to be further developed or even for new designs to be created. All of this leads to information being lost or added over the course of any process, which leads to the said differences. Therefore, efforts were made to standardise to a certain extent the data that was collated. Emphasis was placed on the standardisation of model names, 3D-printed firearm categories, calibres and the names of the people of interest, with a view to facilitating the production of intelligence.

Among the *Website IDs* were also pages whose access was restricted by a paywall or by geo-blocking. Although the data could not be downloaded here, it was possible to retrieve the most important data, such as the name of the model, category, calibre, date of publication and a publisher. However, it was impossible to examine more closely the various files included in the downloads and to determine other relevant information (e.g., about the printing process or other implicated people).

The dataset we have been able to aggregate is substantial, but it is not exhaustive given the widespread distribution of the community on the internet. Nevertheless, this project has resulted in the compilation of a considerable amount of data, from a wide range of sources, which are considered to be primary sources among the members of the 3D-printed firearm community. We therefore believe that this dataset provides a useful and valid basis for gaining insight into current trends, developments, and categories of 3D-printed firearms that law enforcement is likely to encounter at a scene of investigation.

### 3.2 Sources and categories of 3D-printed firearms

**Fig. 2:**
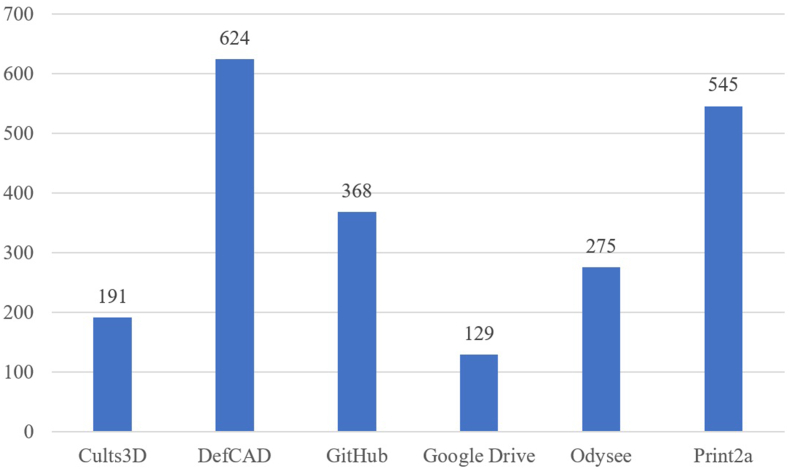
Number of different 3D-printed firearms per Website ID. Thingiverse was not included in this graph due to the low number of 3D-printed firearm blueprints found on this website (n = 1).

First of all, a focus was put on the *Website IDs* where the ‘plans’ of 3D-printed firearms can be found. In this paper, plans refer to a package of files that are included in the download of a 3D-printed firearm. This includes, amongst others, the actual blueprints in a variety of formats (e.g., STL, STEP, OBJ, etc.), manufacturing instructions, images and videos. In total, seven *Website IDs* were consulted during this data collection. The number of entries per *Website ID* is visualised in Fig. 2. On Thingiverse, which is a dedicated website for the sharing of 3D plans of any objects, only one 3D-printed firearm blueprint was found during the whole time. This item was a shotgun of the hybrid firearm category. It has since been removed due to the site policy. This can be viewed as an example of how this data is not always stable over time on the internet. Due to the low number of files found, Thingiverse was not included in the aforementioned figure. On most sites the plans could be downloaded for free, while on other sites the access was restricted. Access to the plans on DEFCAD has been denied due to geo-blocking, as well as being paid access [1]. Furthermore, registration on DEFCAD must be fulfilled with a social security number available only to US citizens. Cults3D is a site like Thingiverse, but with a less strict policy where 3D-printed firearm blueprints can be offered for free or against payment [13]. In the case of entries from DEFCAD or Cults3D, which account for almost 40 % of all entries, less information could be obtained than from the other sources due to the limited access, but it was still possible to examine the critical criteria for this project. Regarding timeliness, the DEFCAD, Odysee, Print2a and Cults3D are continually updated while Google Drive and Github have not been updated for several years. Regarding the *Pathway IDs*, 45 were found among the seven *Website IDs*. 22 of these are on Odysee, which is currently considered the most active source. This may be due to the site's policy which allows easy sharing of files and free access.

**Fig. 3:**
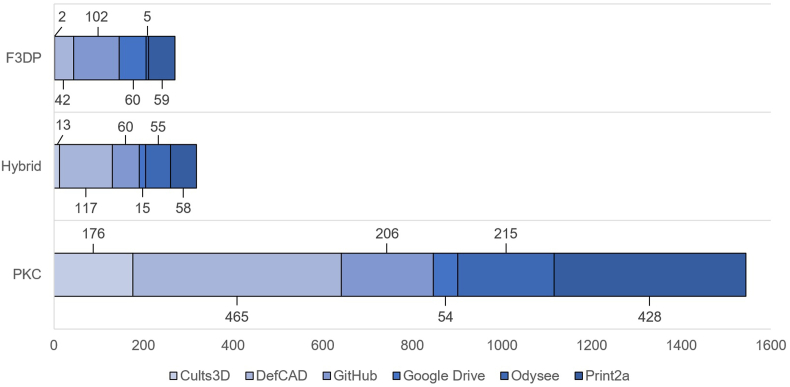
Visualisation of blueprints according to the category of 3D-printed firearms per Website ID. The three categories of 3D-printed firearms are F3DP (n = 270), hybrid firearms (n = 318) and PKC (n = 1544). Thingiverse has been excluded in this graphic due to the low number of 3D-printed firearm blueprints found.

**Fig. 4:**
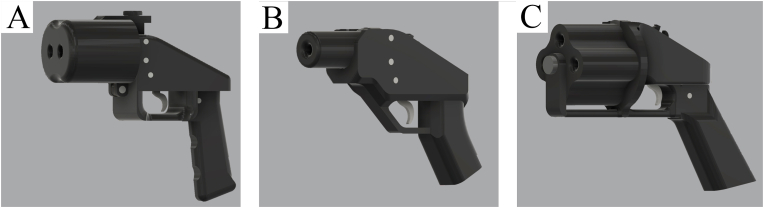
Different F3DP designs of the Liberator. (A) Baby Biden Pistol; (B) Compact Liberator Frame; (C) Pepperbox Liberator v1.1. The renderings were created using files downloaded from Print2a.

Fig. 3 shows the distribution of blueprints according to the category of 3D-printed firearms per *Website ID*. The F3DP category is the smallest containing 270 entries in the data table, with about 85 of these classified as unique designs. For the probably best-known F3DP firearm, the Liberator, a dozen different variants exist which differ in certain specifications (e.g., different calibres, number of barrels) or aesthetic aspects. Three of these variants are shown in Fig. 4. Most of these F3DP designs are handgun-like and are chambered in less-powerful calibres as the pressure bearing parts are made of polymer and do not withstand the pressure of larger calibres or repeated firing.

**Fig. 5:**
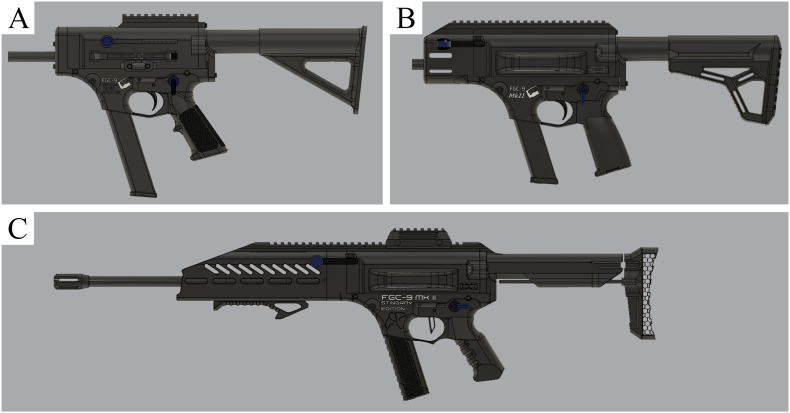
Different versions of the FGC-9 (9 × 19 mm Parabellum). (A) FGC-9 MKI; (B) FGC-9 MKII; (C) FGC-9 MKII Stingray. The rendering was created using files downloaded with the FGC-9 plans from Odysee.

As for the category of hybrid firearms, a total of 322 entries were recorded. As it was the case throughout this study, we were faced with the problem that designations can vary from one source to another for the same 3D-printed firearm model. Reducing to a basic model was not always straightforward given the different information available per source. Remixes of already existing designs are much rarer in this category compared to PKC designs. Here, a remix is considered to be a variation of an existing model that has essentially aesthetic changes which do not alter the basic model. An improved version of an existing model is not considered a remix if it contains essential design changes. In total, about 170 unique hybrid designs have been collated. Hybrid models can be upgrades of existing F3DP firearms (e.g., the Liberator with a Glock barrel), but more often they are newly designed 3D-printed firearms. The selection ranges from small pistols to submachine guns or even shotguns. Arguably the most famous design is the FGC-9 (9 × 19 mm Parabellum), whose name stands for “Fuck Gun Control” and which was published for the first time in 2020 [8]. This 3D-printed firearm embodies the fundamental principles on which much of this community is based: the right to defend one's freedom and the right to own firearms. The FGC-9 gained much attention when reports emerged from the civil war in Myanmar showing its alleged use in combat [14,15]. Since its appearance on the internet, improved versions (internal and external modifications) have also been published (see Fig. 5).

**Fig. 6:**
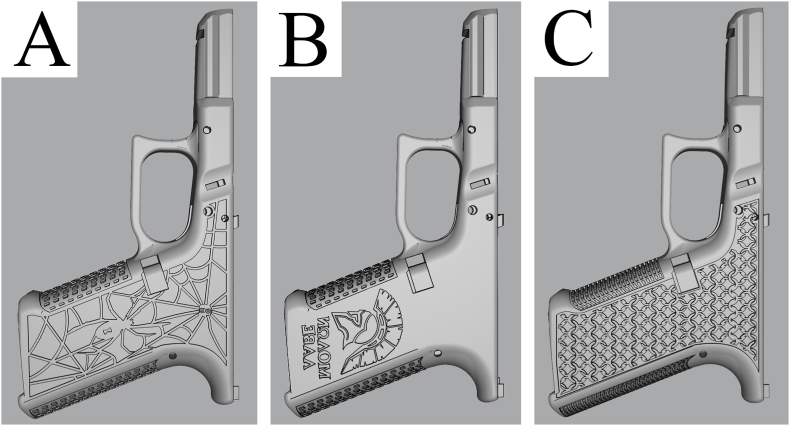
PKC designs of different Glock 19 frame remixes published by the Blacklotus Coalition. These designs are based on the same model, but the aesthetic aspect changes. (A) Spider theme; (B) Spartan theme; (C) Chain theme.

The number of PKCs amounts to 1,544 and clearly separates itself from the other two categories. PKC designs are 3D-printed receivers or frames for firearms, and they include a large number of remixes. For example, a total of 147 entries were collated based on the model FMDA 19.2, a frame for a Glock 19. Fig. 6 illustrates three Glock 19 frames (remixes) designed by the Blacklotus Coalition and downloaded from the website Print2a. Again, given the different information available per source, it made it difficult to reduce similar designations to a basic model. For example, in an apparently similar PKC design of a Glock 17 frame, several different designations could be found: Glock 17, Glock 17.2, Glock DD17.2, Glock 17 P80. This also applies to a large number of other designs. After grouping the different remixes together, the number of unique PKC designs was over 750. Entries in this category mainly include receivers for AR-based rifles (AR-15, AR-22 and AR-10) and frames for Glock pistols (Glock 17, 19 and 26).

In general, a relatively high number of duplicates between the different sources has been observed. It is therefore apparent that the same plans of 3D-printed firearms as well as (almost) the same information can be found in more than just one source. For example, the original Liberator and its different variants have been found in more than ten different *Pathway IDs*, totalling over 70 entries.

**Fig. 7:**
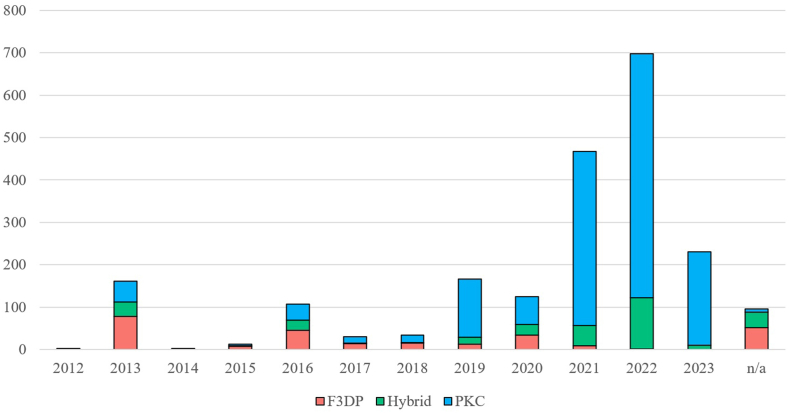
Number of blueprints according to the category of 3D-printed firearms published per year on the different sources. Note that the data collection has been finished in April 2023, explaining the sudden decrease in 2023 in this graph.

Fig. 7 shows the chronological trends of the three categories of 3D-printed firearms plans published on the various sources over the last decade. It was sometimes difficult to assign a precise release date to each entry, either because the information on the website was missing or because nothing could be found in the files themselves. Due to the diverse information between the sources, it often occurred that different publication dates were entered for certain 3D-printed firearm models. This demonstrates once again the volatility of information between different sources. Therefore, it is important to note that this figure does not reflect the actual release date of the 3D-printed firearms but the publication date on the different sources. So, it shows the activity related to the different categories of 3D-printed firearms.

Fig. 7 clearly shows an acceleration of the activity and an increase in the number of publications in the last few years. This trend is largely due to the PKC designs which increased strongly in 2019 before declining somewhat in 2020. This trend was followed by another massive increase, due to the releases of many remixes of already existing PKC firearms. Releases of hybrid firearms have also increased steadily in recent years, but to a lesser extent than PKC. Ultimately, F3DP experienced a final surge in 2020 before their numbers plummeted over the last two years. The decline of the F3DP category is mainly due to the lower performance and reliability of this category of 3D-printed firearms, which eventually led to a shift towards hybrid designs.

**Fig. 8:**
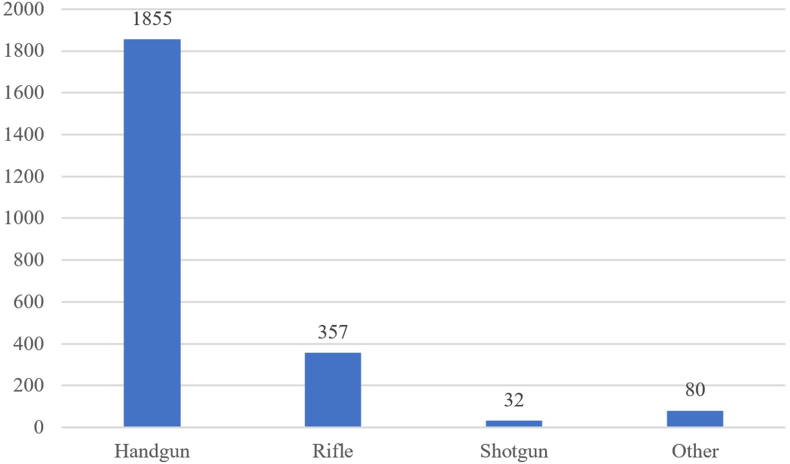
Number of 3D-printed firearm blueprints per calibre type. Note that there are more calibres than entries in the database. This is due to the 3D-printed firearms that can be built in various calibres.

Regarding the calibres, a total of 31 different calibres have been collated (see Appendix, Table A1). In around 1 % of the cases, it was not possible to assess the calibre to a 3D-printed firearm blueprint due to the lack of information. The calibres were grouped into four different types: (i) Handgun (e.g., .22 Long Rifle, 9 × 19 mm Parabellum, .380 ACP or .45 ACP), (ii) Rifle (e.g., 5.56 × 45 mm NATO, 7.62 × 51 mm NATO or 7.62 × 39 mm) (iii) Shotgun (i.e., .410 Bore or 12 Gauge), and (iv) Other. The distribution of these calibre types is depicted in Fig. 8. The vast majority of calibres fall into the “Handgun” type. One reason for this is that F3DP and hybrid firearms are inferior to real firearms in terms of durability and would not withstand the pressure of larger calibres or the repeated discharge of high-power calibres. That said, some hybrid firearms are designed in very surprising calibres with high pressure ranges, such as the Russian 7.62 × 39 mm calibre or shotgun compatible 12 Gauge ammunition. Although there has been positive feedback about these on social media (especially Twitter and Reddit), questions and concerns about the reliability of such 3D-printed firearms in the long run remain. F3DP are largely designed for calibres with a projectile diameter of around 6 mm (e.g., .22 Long Rifle or 6.35 × 15.5 mm SR Browning, etc.). These cartridges release lower pressure during the discharge meaning that the polymer structure may better resist. Hybrid firearms often use the 9 × 19 mm Parabellum calibre, as these 3D-printed firearms are considered more durable and may absorb more pressure than F3DP designs [8]. There is also a large proportion of PKC frames that are used in pistol builds and therefore use handgun calibres as well. In the “Rifle” type, the 5.56 × 45 mm NATO calibre is particularly prominent, as many PKC builds are based on the lower receiver of the AR-15. Calibres for shotguns as well as the remaining calibres that cannot be clearly classified made up the smallest share. The extremely large calibres 26.5 mm and 37 mm are intended for signalling devices (e.g., flare guns) whose ammunition is available in such calibres. It was not possible to attribute a calibre in 23 cases due to missing information in the sources.

### 3.3 People of interest

**Fig. 9:**
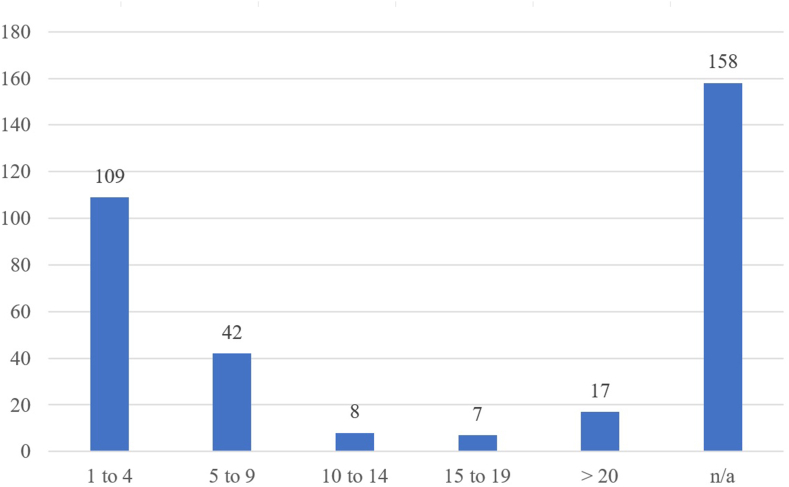
Number of occurrences of main PoI per number of attributed designs released. “n/a” designates the number of designs for which no PoI could be assigned.

People of interest (PoI)^2^ were searched for, who were either involved in the design or the publication. Due to the information available as well as the differences between the various sources, it was not always possible to assign a person to an entry or to distinguish between the designer of the model and the publisher. But the compilation of this data provides an indicator of the activity of different persons across the different sources. Fig. 9 shows the distribution of the number of entries that have been attributed per PoI. Most of the people are associated with one to four entries, but at the same time there is a significant number of entries that cannot be attributed to anyone (n/a). There are also some people whose names are found on a larger number of entries. For example, the two most frequent names are associated with 232 and 332 occurrences respectively, and they are from designers who mainly developed PKC remixes. Most of the names which occurred more than 20 times are associated with PKC designs, with the exception of 4 of them who are designers focusing on F3DP and hybrid firearms. In general, names that appeared more than 10 times are individuals who are well-known and well-connected within the 3D-printed firearms community. As can be read in posts or in feedbacks on various platforms (e.g., Twitter, Reddit), their work is highly valued.

We also included the variable called “Other PoI”, which was used to record names of individuals that could also be associated with the 3D-printed firearm in question. These people mostly contributed to designing and testing the 3D-printed firearm or were serving as consultants due to their experience. The community is now so well networked and established that designs to be released are first beta tested. In these tests, selected people evaluate a 3D-printed firearm plan and provide feedback for improvement before the plans are published on the internet. Individuals appearing here were either already known as main PoI, or names that had not appeared before. It is not uncommon for the same names to appear several times in this variable, showing the commitment and dedication of some people in this field.

### 3.4 Manufacturing instructions

In just almost 1,100 entries, it was found that some kind of instruction was available. The content of these instructions varied from one blueprint for a 3D-printed firearm design to another, but they may have contained information about the manufacturing process (printing and assembly) as well the names of people involved in the design or testing. In almost three quarters of the cases, these were in the format of simple text files (.TXT), no more than a few pages long. The remaining manuals were in the form of PDF files, mainly in 3D-printed firearm blueprints that were published since 2019. These PDF files can be dozens of pages in size. The FGC-9 MKII, for example, includes a manual that is 194 pages long and contains detailed information for each step of the manufacturing process. In addition to these files, images and videos were sometimes linked, showing either assembly tutorials or test firings.

Information on the printing material is also found in the manuals, but sometimes also in the descriptions on the websites. In over 900 entries it was possible to find details about the recommended material. PLA and other PLA variants were the most frequently suggested polymer. Especially PLA+ from eSun, which is characterised by enhanced mechanical properties and an ease of use, has been suggested more and more often in recent years. Published police reports on crimes involving 3D printed firearms show that this material is indeed used. Examples are the dismantled fabrication sites of 3D-printed firearms in the Netherlands as well as in Finland in 2021 [16,17]. Among others, polymers such as ABS, nylon and PETG are also mentioned for manufacturing, but with less frequency. These polymers may offer better mechanical properties but are more difficult to use in a 3D-printing process.

## 4 Conclusion

Since the publication of the first Liberator blueprint in May 2013, there have been many proposed developments in 3D-printed firearms, and several communities have formed to expand and facilitate the dissemination of information on the internet (particularly on the Clear Web) for the production of such homemade firearms, whole or in parts. The more recent designs such as the hybrid firearms and PKC builds are deemed to be more reliable and durable than the first released F3DP firearms, attracting even more interested people, including ones with criminal intentions. In the face of this frenetic development and the risks it entails, we are convinced that it is necessary to structure and consolidate knowledge about the variety and availability of these homemade firearms on the internet. This is a necessity that is enhanced by the lack of published studies, criminal statistics and law enforcement communication [10,18].

In this research, online content on the development and production of 3D-printed firearms and their components have been searched, indexed and collected. The relevant online content on the Clear Web have been saved and the collected data (i.e., plans: blueprints, instructions, images, videos, etc.) have been collated and their information structured in an Excel spreadsheet. Data about more than 2,100 3D-printed firearm blueprints was collated, composing an unprecedented body of knowledge to help understand the way in which these homemade firearms have developed on the internet. Seven *Website IDs* have been determined, which are comprised of 45 *Pathway IDs*. Most of these sources are accessible without restrictions, reflecting the mindset of the community which supports open access across the world to these files. Trends in recent years have shown an overall increase in activity as well as an increased interest in hybrid firearms and PKC builds. The fact that these two categories of 3D-printed firearms are considered more reliable and perform better certainly affected this increased interest. Most F3DP firearms are designed for handgun calibres, where especially the .22 Long Rifle calibre is common due to less risk of damage. However, hybrid designs and PKC builds are generally designed for withstanding more powerful handgun ammunition including the 9 × 19 mm Parabellum calibre or even rifle cartridges. In the future, the development of these 3D-printed firearms can be expected to continue, delivering even more reliable and performant designs to users. Therefore, this research analysed the taxonomy of 3D-printed firearms and their components accessible on the internet, while providing a structured multimedia collection of information related to the development and production of these homemade firearms. This systematic classification, grouping and structuration of the recorded data supported the identification of patterns of the main threat trends related to 3D-printed firearms, and thus provides valuable information on models of 3D-printed firearms and their components likely to be encountered during crime scene investigations, criminal investigations or customs inspections.

For the future, the monitoring of blueprints of 3D-printed firearms on the internet will continue and future release of new designs will be added to our directory. In this way, knowledge about the variety and availability of the different models will be kept up to date. As mentioned before, it seems important that law enforcement agencies are aware of the categories of 3D-printed firearms they can deal with. Therefore, for a future project, a database could be created that lists the 3D-printed firearm and, all related information and files to which authorities have access. Thus, further projects around the exploitation of data on 3D-printed firearms on the internet will have to be carried out in order to provide more knowledge to law enforcement agencies which can be used in the investigation of cases involving such firearms.

## CRediT authorship contribution statement

**Stefan Schaufelbühl:** Conceptualization, Methodology, Formal analysis, Validation, Investigation, Writing – original draft, Writing – review & editing, Visualization, Supervision, Project administration. **Aurélie Szwed:** Conceptualization, Methodology, Formal analysis, Investigation, Writing – original draft, Visualization. **Alain Gallusser:** Conceptualization, Methodology, Validation, Investigation, Writing – original draft, Writing – review & editing, Supervision, Project administration. **Olivier Delémont:** Conceptualization, Methodology, Validation, Investigation, Writing – original draft, Writing – review & editing, Supervision, Project administration. **Denis Werner:** Conceptualization, Methodology, Formal analysis, Validation, Investigation, Writing – original draft, Writing – review & editing, Visualization, Supervision, Project administration.

## Declaration of competing interest

None.

## Figures and Tables

**Table A1:** List of entered 3D-printed firearm blueprints per calibre type. Note that the calibre of 23 3D-printed firearm blueprints could not be determined.

Handgun		Rifle		Shotgun		Other	
Calibre	#	Calibre	#	Calibre	#	Calibre	#
.22 Blanks	6	5.56 × 45 mm NATO	264	.410 Bore	13	GyroJets	4
.22 Long Rifle	313	7.62 × 51 mm NATO	54	12 Gauge	19	.177 calibre	8
.22 Short	18	7.62 × 39 mm	20			3.5 mm	1
6.35 × 15.5 mm SR Browning	9	.300 BLK	2			5 mm	1
7.65 × 17 mm SR Browning	38	.308 Winchester	16			6 mm	21
7.62 × 25 mm Tokarev	7	.50 BMG	1			26.5 mm	11
9 × 19 mm Parabellum	1141					37 mm	11
9 × 18 mm Makarov	7						
.357 Magnum	21						
.380 ACP	89						
.38 Special	22						
.38 S&W	20						
.40 S&W	100						
.44 Special	15						
.45 ACP	34						
.45 Long Colt	7						
10 × 25 mm Auto	8						

## Footnotes

1 The full table of data could be made available to interested parties, such as law enforcement agencies or forensic researchers, on request from the authors.

2 It was decided not to disclose the pseudonyms of the PoI in this research and to anonymise them.
